# Prevalence and predictors of solid or hematological malignancies in a monocentric cohort of HIV patients from central Italy

**DOI:** 10.7448/IAS.15.6.18084

**Published:** 2012-11-11

**Authors:** E Mazzotta, A Agostinone, F Sozio, T Ursini, E Polilli, M Tontodonati, E Tracanna, G Placido, A Pieri, A Consorte, P Cacciatore, F Di Masi, G Calella, S Falorio, M Leva, M Vizioli, F Angrilli, L Manzoli, G Parruti

**Affiliations:** 1 University “G. D'Annunzio”, Chieti, Italy; 2 Pescara General Hospital, Infectious Disease Unit, Pescara, Italy; 3 Pescara General Hospital, Microbiology Unit, Pescara, Italy; 4 Pescara General Hospital, Hematology Unit, Pescara, Italy; 5 Pescara General Hospital, Oncology Unit, Pescara, Italy; 6 University “G. D'Annunzio”, Public Health and Biostatistic, Chieti, Italy

## Abstract

**Introduction:**

HIV-infected patients have a higher risk of developing cancer than the general population. Kaposi's sarcoma (KS), non-Hodgkin's lymphoma (NHL), primary CNS lymphoma (PCL) and invasive cervical cancers are considered AIDS-defining. An increased incidence in recent years, however, has been reported also for other malignancies after the introduction of HAART.

**Methods:**

We performed a case-control study to characterize all HIV-infected patients with both AIDS and non-AIDS-defining neoplasms observed among all consecutive patients followed at the Infectious Diseases Unit of Pescara General Hospital, since 1991 through 2012. All cases were matched with equinumerous controls without neoplasia homogeneous for age, sex and AIDS diagnosis.

**Results:**

Out of 626 patients consecutively assisted since 1991, 57 cases of malignancy (9.1%) were observed. Of these, 45 (79.0%) occurred in males; mean age was 43.6±9.3 years; 49 (86.0%) patients were diagnosed with AIDS. Tumors observed were: NHL, 17 (29.8%); SK, 13 (22.8%); HCC, 5 (8.8%); CPL, 6 (10.5%); Hodgkin's lymphoma, 4 (7.0%); solid tumors, 12 (21.1%), including 1 AIDS-defining tumor (anal cancer). Among these, 37 (66.1%) patients died; of them 14 (37.8%) had non-AIDS cancers. Cases were well matched with the 55 controls for sex (p=0.9), age (p=0.6) and AIDS diagnosis (p=0.6). In comparison with controls, CD4 nadirs were not different (153±151 in controls vs 136±154 cells/mmc), while CD4 at tumor diagnosis were very different between controls (463±283 cells/mmc) and cases (226±209 cells/mmc, p<0.0001). Among patients with malignancies, those who died had a non-significant reduction in CD4 counts (p=0.14); seemingly irrelevant were smoking status (p=0.9), working ability (p=0.4), HCV coinfection (p=0.4). Surprisingly, in patients co-infected with HBV, including HBsAg negative, antibody-positive subjects, tumors were significantly more frequent (60.7% vs. 38.8%, p=0.009).

**Conclusion:**

Factors potentially relevant for carcinogenesis in the prolonged survival patients of the HAART era may include HBV coinfection in spite of the lack of active biochemical activity (HbsAg negative) in the majority of coinfected patients. The potential relevance of this finding deserves prompt assessment in a larger multicentric cohort.
